# Microencapsulation of Horseradish (*Armoracia rusticana* L.) Juice Using Spray-Drying

**DOI:** 10.3390/foods9091332

**Published:** 2020-09-21

**Authors:** Lolita Tomsone, Ruta Galoburda, Zanda Kruma, Vanessa Durrieu, Ingmars Cinkmanis

**Affiliations:** 1 Department of Food Technology, Faculty of Food Technology, Latvia University of Life Sciences and Technologies, Rigas Street 22, LV-3004 Jelgava, Latvia; ruta.galoburda@llu.lv (R.G.); zanda.kruma@llu.lv (Z.K.); 2 Laboratoire de Chimie Agro-industrielle, LCA, Université de Toulouse, INRAE, F-31030 Toulouse, France; vanessa.durrieu@ensiacet.fr; 3 Department of Chemistry, Faculty of Food Technology, Latvia University of Life Sciences and Technologies, Rigas Street 22, LV-3004 Jelgava, Latvia; ingmars.cinkmanis@llu.lv

**Keywords:** microencapsulation, antioxidant activity, wall material, core-to-wall ratio, storage stability

## Abstract

Horseradish contains many bioactive compounds with antioxidant activity. The current study aimed to evaluate the effect of various wall materials and their ratios on the physical properties and bioactive-compound retention and stability in microencapsulated horseradish leaf and root juices. Horseradish juice was microencapsulated using maltodextrin, maltodextrin/gum Arabic, soy protein isolate, and starch with three different core-to-wall ratios. The total phenolic, total flavonoid, total flavan-3-ol, and total phenolic-acid contents, as well as antioxidant activity, were determined using spectrophotometric methods, whereas individual phenol profiles were determined by high-performance liquid chromatography (HPLC). Multivariate analysis of variance showed that plant material, wall material, and core-to-wall ratio had a significant effect on the bioactive-compound retention and antioxidant-activity preservation. Microcapsules produced from horseradish leaf juice had a significantly higher content of phenolic compounds and antioxidant activity compared to root-juice microcapsules. However, better retention was observed for microencapsulated horseradish root juice. Maltodextrin and maltodextrin/gum Arabic were the most effective wall materials for the retention of bioactive compounds, while they also had a smaller particle size and better solubility. The horseradish-juice microcapsules possess a high content of rutin. The highest stability of bioactive compounds after storage was found at a core-to-wall ratio of 20:80.

## 1. Introduction

Horseradish (*Armoracia rusticana* L.) is a perennial herb of the Brassicaceae family, widely used in culinary and folk medicine. A recent study by Dekic et al. [1] confirmed the functionality of both aboveground and underground parts, demonstrating the in vitro and in vivo effects of horseradish pure constituents. Brassicaceae family plants contain various bioactive constituents, such as phenolics [2], enzymes (peroxidase, myrosinase, and glucosinolates) [3], and other compounds, giving rise to their antioxidant and anticarcinogenic activity [4].

Following the concept of “green chemistry”, the best method of compound extraction would involve obtaining juice without any solvents. However, the use of juice is limited due to enzyme activity and the degradation of compounds caused by various internal and external factors. As a result, the biological value of horseradish juice decreases with storage time. Various technologies were developed for vegetable-juice preservation; however, they typically result in a loss of bioactive compounds due to thermal or oxidative reactions [5]. Recently, microencapsulation was proposed as the final processing step to stabilize bioactive compounds. According to a review by Labuschagne [6], the most popular encapsulation methods over the last 20 years were spray-drying and freeze-drying, which require water-soluble wall materials. Several studies confirmed that spray-dried encapsulated products had higher moisture content, lower solubility, and higher hygroscopicity compared to freeze-dried products [7]. Additionally, spray-drying provided a higher phytochemical content in blueberry polyphenol–protein matrices [7].

A large variety of materials can be used for the encapsulation purposes [6]. Polysaccharides (such as maltodextrin, modified starches, chitosan, gum Arabic, and alginates), proteins, and lipids, as well as their mixtures, are commonly used. Many studies demonstrated the impact of wall material on phytochemical stability. Gum Arabic is very viscous [8]; therefore, it is often used in combination with maltodextrin. Zhang et al. [9] revealed a better retention of phenolics in cranberry juice when blended wall material (gum Arabic/maltodextrin) was used, compared to single materials. Similarly, Idham et al. [10] demonstrated the best productivity and efficiency rate for combined maltodextrin and gum Arabic (6:4, *w*/*w*), indicating the importance of the wall-to-core ratio. However, efficiency is highly dependent on the food matrix, the type of phenolic compound encapsulated, and the coating composition [11].

The size and distribution of particles obtained in the spray-drying process vary in a wide range [12,13]. They are greatly affected by the wall material and its interaction with the core material. Particle size, solubility, and hygroscopicity are important attributes for the further application of powders [12]. The use of different wall materials in the microencapsulation process results in various physicochemical properties depending on the structure and properties of specific materials.

The current study aimed to evaluate effect of various wall materials and their ratios on the physical properties and bioactive-compound retention and stability in microencapsulated horseradish leaf and root juice. The total phenolic compounds, total phenolic acids, total flavonoids, and total flavan-3-ols, as well as antioxidant activity, were studied.

## 2. Materials and Methods

### 2.1. Plant Materials and Encapsulating Agents

Fresh horseradish (*Armoracia rusticana* L.), which was used for juice production, was collected in Latvia (latitude: 56°39′ N, longitude: 23°44′ E); the leaves were collected in July 2018, while the roots were collected in November 2018. Plant material was frozen and stored at −18 °C until further use. Horseradish root and leaf juices were obtained by grinding the frozen samples before extraction in a basket press. The obtained juice was freeze-dried to 93% dry matter and used in further studies.

Some wall materials used for encapsulation, i.e., maltodextrin (MD) with a dextrose-equivalent value (DE) of 12, gum Arabic (G), and soluble starch (S), were purchased from Sigma-Aldrich (St. Louis, MO, USA). Soy protein isolate (SPI), 90% pure, was purchased from Lustrel Laboratoires SAS (Saint Jean de Vedas, France).

### 2.2. Chemicals and Reagents

All chemicals were of analytical or high-performance liquid chromatography (HPLC) grade. Gallic acid, Folin–Ciocalteu phenol reagent, and 2,2-diphenyl-1-picrylhydrazyl (DPPH^•^) were purchased from Sigma-Aldrich (Buchs, Switzerland). All other chemicals used in the research were obtained from Acros Organics (Belgium, WI, USA).

### 2.3. Infeed Solution Preparation

Different core-to-wall ratios of 20:80, 50:50, and 80:20 with various wall materials were prepared for the study (Table 1). The infeed solution contained 5% (*w*/*w*) total solids.

The wall material and freeze-dried juice were dissolved in deionized water.

Maltodextrin and maltodextrin/gum Arabic with juice were mixed at room temperature for 30 min under constant mechanical stirring (1000 rpm). Soy protein isolate with juice was stirred for 30 min at 70 ± 2 °C at 5000 rpm and further held in the same conditions during spray-drying.

Starch was dissolved in deionized water at room temperature for 30 min under mechanical stirring at 1000 rpm; then, after emulsion, it was heated at 90 ± 2 °C for 30 min under mechanical stirring (1000 rpm), in order to allow maximum starch solubilization. Then, the starch solution was left overnight at room temperature. The next day, the starch solution with juice was mechanically stirred (1000 rpm) at room temperature for 30 min.

The total amount of prepared solution for each sample was 500 g; each experiment was performed in triplicate.

### 2.4. Encapsulation of Horseradish Juice by Spray-Drying

Freshly prepared infeed solutions were spray-dried in a Mini Spray Dryer B-290 (Büchi, Flawil, Switzerland) under the following stable process conditions: inlet air temperature, 120 ± 4 °C; outlet air temperature, 80 ± 4 °C; drying airflow rate, 470 L/h; liquid feed flow rate, 0.33 L/h; aspiration, 100%. During the spray-drying process, solutions were mixed with a magnetic stirrer (magnet size 4.0 × 0.5 cm) at 500 rpm at room temperature (20 ± 1 °C), except for the solutions with SPI. All infeed solutions with SPI during the spray-drying process were mixed with a magnetic stirrer (magnet size 4.0 × 0.5 cm) at 500 rpm at 70 ± 1 °C. Microcapsules were collected in the cyclone collector, then sealed in hermetic packaging, and stored for further analysis in the dark at room temperature.

Spray-dried juices without added materials were used as the control (blank).

### 2.5. Measurement of Physical Parameters

The microcapsule size in dry experimental powder was determined on the basis of the scattering pattern of a transverse laser light using Scirocco 2000 equipment (Malvern Instruments, Worcestershire, UK). The mean particle diameters were determined ranging from 0.2 to 2000 μm. The used refractive index was 1.52, the air pressure of dispersion was 4 bars, and the degree of vibration was 70%. The volume-based particle diameter was calculated as the mean of three measurements per sample.

For hygroscopicity measurements, 1 g of powder was placed in a container at 25 °C with a saturated NaCl solution, obtaining 75.29% relative air humidity [14,15]. Samples were weighed after one week. Hygroscopicity was evaluated on the basis of the moisture absorption capacity, expressed as grams of absorbed moisture per 100 g of dry matter.

Solubility was determined according to the method proposed by Cano-Chauca et al. [16], with some modifications. One gram of sample was mixed with 100 mL of distilled water, and the mixture was stirred with a magnetic stirrer (MS01) for 30 min. Then, the solution was centrifuged (ELMI CM, Riga, Latvia) at 3500 rpm for 5 min. A 25 mL aliquot of the supernatant was transferred to a preweighed Petri dish and immediately oven-dried in a Universal Oven UF55 (Memmert GmbH+Co.KG, Germany) at 105 °C for 5 h. The solubility was calculated using the weight difference and expressed as a percentage (%).

### 2.6. Calculation of Retention Efficiency (RE) and Stability

The RE of each biologically active compound was calculated using Equation (1).

RE (%) = (Active compound in the microcapsules/Active compound in the infeed solution) × 100%(1)

Microencapsulated horseradish-juice powders were packaged in hermetically sealed plastic containers and stored at room temperature (22 ± 1 °C) in the dark. The stability of biologically active compounds after four months of storage was calculated using Equation (2).

Stability (%) = (Active compound after storage/Active compound before storage) × 100%(2)

### 2.7. Chemical Properties of Encapsulated Horseradish-Juice Powder

For all samples, the moisture content was determined in triplicate according to the standard ISO 6496:1999, and all results were expressed on the basis of dry weight (DW).

#### 2.7.1. Spectrophotometric Determination of Bioactive Compounds and Antioxidant Activities

For the extraction procedure, 0.1 g of powder was dissolved in 100 mL of distilled water with the assistance of a magnetic stirrer (magnet size 4.0 × 0.5 cm) at 700 rpm for 20 min at room temperature (20 ± 1 °C). Spectrophotometric analysis was completed using a JENWAY 6300 (Barloworld Scientific Ltd., Staffordshire, UK).

Total phenolic content (TPC) was determined with the Folin–Ciocalteu spectrophotometric assay [17] in mg of gallic-acid equivalent (GAE)/100 g DW. TPC was calculated using a standard curve of a gallic-acid solution in water at various concentrations (0.01–1 mg/mL).

Total phenolic-acid content (TPAC) was determined using a spectrophotometric method [18], expressed as mg of caffeic-acid equivalent (CAE)/100 g DW. TPAC was calculated from the standard curve, which was constructed for a standard aqueous solution of caffeic acid with a concentration between 0.001 and 0.3 mg/mL.

Total flavonoid content (TFC) was determined according to a colorimetric method [19] with modifications described by Blasco et al. [20], and it was expressed as mg of (+)-catechin equivalent (CE)/100 g DW. TFC was calculated according to the standard curve of a (+)-catechin aqueous solution at concentrations of 0.01–0.4 mg/mL.

Total flavan-3-ol content (TF3C) content was determined using Zam et al.’s [21] method, expressed as mg of (+)-catechin equivalent (CE)/100 g DW using a standard curve established at various concentrations (0.1–12.0 mg/mL) of a (+)-catechin solution in water.

The 2,2-diphenyl-1-picrylhydrazyl (DPPH^•^) radical-scavenging activities were determined according Yu et al.’s method [22]. The radical-scavenging activity of the extract was also measured using the 2,2′-azino-bis(3-ethylbenzo-thiazoline-6-sulfonic) acid (ABTS^•**+**^) radical cation assay, described by Re et al. [23]. Both antioxidant activities were expressed as mmol of Trolox (6-hydroxy-2,5,7,8-tetramethylchroman-2-carboxylic acid) equivalent (TE)/100 g DW. Radical-scavenging activities were calculated according to the standard curve, established at various concentrations of Trolox (0.1–10 mmol/mL). The reducing power (RP) was determined using the method of Athukorala et al. [24]. This parameter was expressed as mg of ascorbic-acid equivalent (AAE)/100 g DW, and calculations were completed according to the standard curve of an ascorbic-acid aqueous solution at concentrations of 0.025–0.5 mg/mL.

#### 2.7.2. HPLC Analysis of Individual Phenolic Compounds

The weight of the microencapsulated samples for extraction was selected to obtain equal dry-matter mass; 0.25 ± 0.01 g of sample was extracted with 25 mL of 1 N HCl/EtOH/H_2_O (1:80:19 *v*/*v*/*v*) in an ultrasonic bath YJ5120-1 (Oubo Dental, St. Louis, MO, USA) at 35 kHz for 10 min at 20 ± 1 °C. The extracts were then centrifuged in a CM-6MT centrifuge (Elmi Ltd., Riga, Latvia) at 3500 rpm for 5 min. The supernatants were combined in a 25 mL graduated flask and filled to the mark with solvent. Separation and quantification were carried out using a Prominence high-performance liquid chromatography system LC-20AD (Shimadzu, Kioto, Japan) with a YMC C18 analytical column and a SPD M20A photodiode array detector according to the procedure described by Priecina et al. [25]. The following standard substances were used for the identification and quantification of phenolic compounds: rutin (Sigma, Guiyang, Guizhou, China), (−)-epicatechin (Sigma, New Delhi, Delhi, India), (+)-catechin (Sigma, Guiyang, Guizhou, China), and sinapic acid (Fluka, Atlanta, GA, USA). The results were expressed as mg per 100 g of dry weight (mg/100 g DW).

### 2.8. Statistical Analysis

The results are presented as the mean value ± standard deviation. Analysis of variance (ANOVA) and Tukey’s multiple-comparison test were carried out to determine differences in parameters among the samples. Differences were considered as significant at *p* ≤ 0.05. Multivariate analysis of variance (MANOVA) was used to evaluate the influence of factors such as plant part, wall material, and core-to-wall ratio on the dependent variables (total phenolic, total flavonoid, total flavan-3-ol, and total phenolic-acid contents, as well as antioxidant activity and individual phenol profiles). Estimated marginal means for each factor were presented. A linear correlation analysis was carried out using SPSS version 17 (SPSS Inc., Chicago, IL, USA).

## 3. Results and Discussion

### 3.1. Physicochemical Characteristics of Microencapsulated Horseradish Leaf and Root Juices

The particle size of the microcapsules have a significant effect on sensory properties (texture, taste, etc.) and physical properties (e.g., solubility and hygroscopicity). The particle size of the obtained microcapsules ranged from 3.64 µm (R50MG50) to 18.40 µm (R20S80) (Table 2). This is in accordance with the results of other studies performed using spray-drying technology [26]. It was found that a smaller particle size results in a larger surface available to water [27], whereas a larger particle size reduces water absorption [28].

The size of the microcapsules was significantly affected by the core material; the horseradish leaf microcapsules were on average 12% larger compared to the root microcapsules. The wall material significantly affected the particle size, whereby samples obtained with maltodextrin (MD) and maltodextrin/gum Arabic (MG) had smaller particles compared to starch (S) and soy protein isolate (SPI) microcapsules.

The core-to-wall ratio significantly (*p* < 0.05) influenced the particle size of the horseradish-juice microcapsules. Rodsamran et al. [29] and Nesterenko et al. [26] concluded that microcapsules with a higher proportion of core material have larger particle sizes. The same trend was observed for horseradish-root-juice microcapsules with starch (S) and horseradish-leaf-juice microcapsules with soy protein isolate (SPI).

The hygroscopicity of horseradish-juice microcapsules was tested to gain insight into their physical stability, as well as their preferred storage conditions. The hygroscopicity of the analyzed samples ranged from 7.46 g of absorbed moisture per 100 g DW (L20S80) to 20.89 g/100 g DW (R80M20) (Table 2). There were no significant differences between the two core materials (leaf or root juice).

The wall material had a significant (*p* < 0.05) effect on the hygroscopicity of horseradish-juice microcapsules. According to Ferrari et al. [30], the hygroscopicity of microencapsulated powder is mainly influenced by the composition of the wall material. The wall materials studied in our experiments had different molecular weights. Akhavan Mahdavi et al. [31] observed that microcapsules with higher-molecular-weight wall materials have lower hygroscopicity compared to microcapsules with lower-molecular-weight wall materials. This trend was also observed for horseradish-juice microcapsules. In the case of horseradish-juice microcapsules, the wall materials could be ranked as follows (starting with the smallest): starch < SPI < MG < MD. Horseradish-juice microcapsules with maltodextrin had more than twofold higher hygroscopicity than microcapsules with starch. Maltodextrin microcapsules with similar hygroscopicity were also obtained by Souza et al. [32] and Tonon et al. [33].

The concentration of the wall material or the wall thickness also affects the hygroscopicity of the microcapsules [30]. The core-to-wall ratio also had a significant (*p* < 0.05) effect on the hygroscopicity of horseradish-juice microcapsules. The results of the study showed a tendency of the hygroscopicity of microencapsulated horseradish juice increasing as the proportion of wall material decreased. De Souza and colleagues made the same observations [32]. In our study, the microcapsules with a core-to-wall ratio of 80:20 had the highest hygroscopicity, while microcapsules with core-to-wall ratios of 50:50 and 20:80 had, on average, 10% and 21% lower hygroscopicity, respectively. Particle size had a strong negative correlation with hygroscopicity (for leaf juice, *r* = −0.822; for root juice, *r* = −0.606).

Solubility is one of the most important functional properties of edible powders. It affects the behavior of the powder when it is reconstituted in water. The solubility of horseradish-juice microcapsules was significantly (*p* < 0.05) affected by both the wall material and the core-to-wall ratio (Table 2). It ranged from 28.80% (R20S80) to 91.02% (L20M80). The water solubility of horseradish-leaf-juice microcapsules was, on average, 7% better than that of horseradish-root-juice microcapsules. In the case of wall materials, better solubility was observed for microcapsules with MG and MD, while significantly (*p* < 0.05) lower solubility was observed for microcapsules with starch. This was consistent with the findings for particle size, where microcapsules with larger particle sizes (such as those using starch (S) and soy protein isolate (SPI)) had lower solubility.

Very good water solubility of microcapsules was also observed by Rezende et al. [34], where the water solubility of encapsulated acerol (*Malphigia emarginata* DC) pulp and residue with GA and MD (DE 9–12) was 99.0%–99.1%, as explained by the properties of wall material used and by the particle size obtained. Specifically, a smaller particle size leads to a larger available surface area for the binding of water molecules, i.e., hydration [27]. Correlation analysis showed a strong negative linear relationship between solubility and particle size for microcapsules of both horseradish leaves (*r* = −0.874) and horseradish roots (*r* = −0.921). A moderate correlation was also established between solubility and hygroscopicity (for leaves, *r* = 0.738; for roots, *r* = 0.531).

Although there were scientists [35] who found that the water solubility of microcapsules is not affected by the core-to-wall ratio or wall material composition, Daza and colleagues [36] concluded that water solubility increased with increasing wall proportion. In our study, the solubility of horseradish-juice microcapsules was affected by the core-to-wall ratio. In general, microcapsules with a core-to-wall ratio of 80:20 had the highest solubility.

In addition, microcapsules with starch and SPI tended to increase the solubility as the proportion of core material increased. In contrast, microcapsules with MD and MG showed the opposite tendency, whereby the solubility decreased with an increasing proportion of core material. This can be explained by the fact that starch has low solubility and high viscosity, whereas MD has high solubility and low viscosity. In this study, the lowest solubility was seen in horseradish-juice microcapsules with SPI, which is similar to the findings of Molina Ortiz et al. [37]. However, the solubility of horseradish-root-juice microcapsules was 28.8%–59.8% and that of horseradish-leaf-juice microcapsules was 46.6%–66.6%. In studies on the microencapsulation of pomegranate juice, it was also observed that solubility was strongly influenced by the type of wall material and, only in some cases, by the core-to-wall ratio [38].

### 3.2. Characteristics of Bioactive Compounds in Microencapsulated Horseradish Leaf and Root Juices

The content of phenolic compounds and the antioxidant activity (AOA) of the horseradish-root-juice and horseradish-leaf-juice microcapsules obtained by spray-drying are presented in Table 3. Among the individual samples, the highest content of TPC was detected for samples L80P20, L80M20, and L80S20, the highest content of TPAC was detected for samples L80MG20 and L80S20, and the highest content of TFC was detected for sample L80P20. The highest DPPH^•^-scavenging activity was confirmed for the horseradish-leaf-juice powder samples encapsulated using the SPI, MG or S as the wall material, with a core-to-wall ratio of 80:20. The highest ABTS^•+^ activity was observed for samples L80P20 and L80S20. Horseradish-root-juice sample R80MG20 had the best reducing power among the studied microcapsule samples.

Similar studies on plant-extract encapsulation proved that the AOA in microencapsulated garlic extract was between 1.15 ± 0.01 and 1.44 ± 0.02 mmol TE/100 g DW [14], the AOA in microencapsulated cranberry juice varied between 3.47 ± 0.1 mmol TE/100 g (maltodextrin with DE 10–13) and 4.89 ± 0.12 mmol TE/100 g of powder (gum Arabic/maltodextrin with DE 10–13) [9], and the AOA in jussara juice ranged from 17.40 ± 1.70 mmol TE/100 g of powder (maltodextrin and oligofructose; 1:1) to 22.43 ± 2.48 mmol TE/100 g of powder (maltodextrin and inulin; 1:1) [39]. These values were significantly lower than those established for horseradish-leaf-juice microcapsules in the current study.

Multivariate analysis of variance showed that plant material (roots and leaves), wall material (MD, SPI, MG, and S), and core-to-wall ratio had a significant effect (*p* < 0.05) on the TPC, TPAC, TFC, TF3C, and AOA on the basis of the three tested methods, namely, DPPH^•^, ABTS^•**+**^, and RP.

#### 3.2.1. Effect of Horseradish Plant Part on Bioactive Compounds in Microcapsules

Microcapsules produced from horseradish leaf juice had a significantly (*p* < 0.05) higher content of phenolic compounds and AOA compared to the root-juice microcapsules (Table 3). This is in accordance with our previous results, where the phenolic content in horseradish leaves was 25-fold higher than that in roots of the same plant [40]. In the current study, the content of phenolic compounds, the DPPH^•^ activity, and the reducing power of the horseradish-leaf-juice microcapsules were almost twofold higher than those in the horseradish-root-juice microcapsules, whereas the ABTS^•+^ activity was almost 12-fold higher. This confirmed that phenolic compounds are not uniformly distributed in all parts of plants [41]; hence, their contents vary in different parts of the same plant [42]. In addition, flavonoids, particularly flavones and flavonols, are strong ultraviolet (UV) absorbers that mainly accumulate in epidermis cells [43]; therefore, their content is much higher in the aboveground parts of the plant.

**Figure 1:**
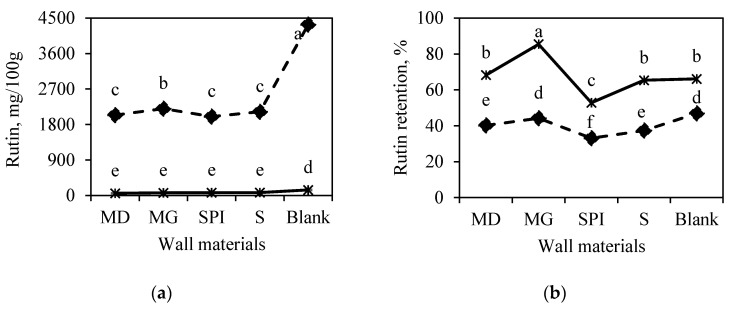

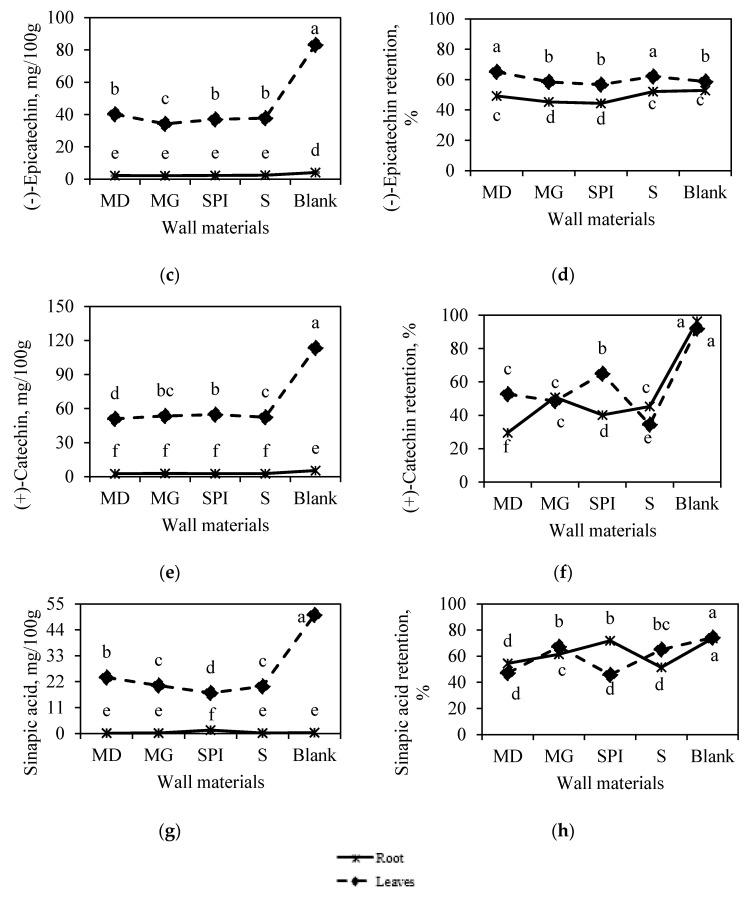
Estimated-marginal-mean plots for the content and retention of individual phenolic compounds: (**a**,**b**) rutin, (**c**,**d**) (-)epicatechin, (**e**,**f**) (+)catechin, (**g**,**h**) sinapic acid in the microencapsulated horseradish juice as a function of the plant part used for juice extraction. ^a–f^ Values with different superscript letters in the same figure are significantly different (*p* < 0.05) on the basis of Tukey’s multiple-comparison test. MD: maltodextrin; SPI: soy protein isolate; MG: maltodextrin/gum Arabic; S: soluble starch; blank: juice powder without wall material.

Among the analyzed individual phenolic compounds (Figure 1a), rutin was the most common phenolic compound in all studied samples; however, in leaf powder, its content was 30-fold higher. Rutin is a well-known flavonoid with a wide range of biological activity. Incandela et al. [44] reported that 500 to 2000 mg of rutin per day is optimal for preventing diseases while maintaining safety from a toxicological aspect. This would be equivalent to 50 g of horseradish-leaf-juice powder (blank) obtained in the current study and 100 g of the respective encapsulated powder. The contents of other phenolic compounds such as (−)-epicatechin, (+)-catechin, and sinapic acid were more than 30-fold lower (Figure 1c,e,g) compared to rutin; moreover, in all samples produced from leaves, their concentration was significantly higher than that in samples made from horseradish roots. Moser et al. [45] obtained grape-juice microcapsules with a (+)-catechin content 31.85–89.63 mg/100 g and an (−)-epicatechin content of 7.15–18.64 mg/100 g, which are similar to our results for horseradish-juice microcapsules.

In all encapsulated samples, the content of phenolic compounds and the AOA were lower compared to the spray-dried juice (blank) due to the included proportion of wall material, which did not contain phenolics, except for the sample with soy protein isolate.

For the evaluation of the microencapsulation process, the retention of compounds in the final product is a very important factor. In our study, the average retention coefficients were higher for root samples (Table 4), except for TFC and TF3C. TPC was significantly reduced after spray-drying, with an RE of 61%–99% for horseradish root juice and 43%–76% for horseradish leaf juice (Table S1, Supplementary Materials). Boyano-Orozco et al. [46] reported an RE of 91%–97% for phenolic compounds in microcapsules of rambutan (*Nephelium lappaceum* L.) peel extract. According to Pang et al. [47], the retention of phenolic compounds in spray-dried *Orthosiphon stamineus* extract was effective, ranging from 66.91% (eupatorin) to 82.08% (rosmarinic acid); however, during microencapsulation by spray-drying, phenolic compounds were lost due to high temperature and the action of oxygen, resulting in their destruction and polymerization [27]. Despite the inevitable losses of phenolic compounds due to the heat treatment, other conformational changes of phenolic compounds may compensate for these losses, as seen for the RE of TPC (99.78% in sample R80S20).

In general, better retention was observed with microencapsulation of horseradish root juice (Table 4). This could be due to the differences in bioactive-compound profile and properties between horseradish root and leaf juice. Indeed, the compounds in horseradish root juice appeared to be more stable in response to environmental factors, including increased temperature during spray-drying.

The retention of rutin was highest in the root juice, especially when using maltodextrin in combination with gum Arabic as the juice wall material (Figure 1b). The opposite trend for (−)-epicatechin was detected with a higher retention in leaf samples (Figure 1d). (+)-Catechin and sinapic-acid retention depended not only on the plant part, but also on the wall material. However, a tendency was not clearly confirmed. It should be noted that, in several cases, the control samples (blank) showed even better retention than the samples with wall materials (Figure 1f,h).

#### 3.2.2. Effect of Wall Material Type on Bioactive-Compound RE

The type of wall material significantly affected the RE (Table 4). Specifically, maltodextrin was the most effective for the retention of TPAC and TF3C, and for DPPH^•^- and ABTS^•**+**^-scavenging activity. MD is the most popular and widely used natural material for microencapsulation. It is a polysaccharide, making it very convenient for use in the microencapsulation process by spray-drying due to its good water solubility. According to Kuck and Noreña [27], polysaccharide wall materials interact with phenolic compounds, forming complex structures and enhancing their stability. In contrast, investigations of *Murraya koenigii* L. leaf extract confirmed MD as the least effective wall material for phenolic-compound retention compared to xanthan and acacia gums [48]. In the current study, the lowest retention using MD was observed for TFC and RP.

The application of maltodextrin combined with gum Arabic resulted in better retention of TPC, TPAC, and TFC, and better RP. These compounds were retained more efficiently because the use of a higher-molecular-weight coating material increased the rate of dry-film formation on the surface of the microcapsule [9]. In a microencapsulation study of tamarillo juice, it was observed that coating materials possessing higher molecular weight (maltodextrin, 41.64 g/mol; gum Arabic, 617.32 g/mol) exhibited greater efficacy for both water- and fat-soluble compounds [8].

Soy protein isolate did not demonstrate the best retention for any of the tested parameters, but it proved to be more effective for preserving TF3C and TFC. SPI has several drawbacks with regard to its application for spray-drying due to its low water solubility and high losses in the drying process. When analyzing the efficacy of SPI for the encapsulation of *Moringa oleifera* leaf extract in terms of TPC, its efficiency was found to range from 52% to 75% [49].

Soluble starch did not achieve the best retention for any of the tested parameters, but higher results for the retention of TPC and TFC were observed. Similar to SPI, soluble starch has several drawbacks for use in the spray-drying process, due to its difficulty in forming a homogeneous suspension compared to the other wall materials analyzed. In the study of Idham et al. [10], soluble starch was found to be the least efficient wall material for encapsulating anthocyanins from *Hibiscus sabdariffa* L.

The combination of MD and GA as a coating material yielded horseradish-root-juice microcapsules with the highest RP and the highest (+)-catechin content, as well as horseradish-leaf-juice microcapsules with the highest TPAC and higher ABTS-cation-scavenging activity (Table 2). Furthermore, higher retention using this wall material was detected for TPC and TFC, in addition to better RP. Similar findings to the encapsulated horseradish leaf juice were reported in jussara (*Euterpe edulis* M.) juice and maltodextrin microcapsules (1229.4 mg GAE/100 g) [39], garlic extract with chitosan/whey protein isolate (11.53–13.87 mg GAE/g DW) [14], and red-grape-skin extract microcapsules (21.43 mg GAE/g DW in samples with 10% polydextrose and partially hydrolyzed guar gum; 25.03 mg GAE/g DW in samples with 10% gum Arabic) [27].

For encapsulation, the wall materials could be ranked in order of their effectiveness in retaining bioactive compounds (starting with the most efficient):

- for horseradish leaf juice, MG > M > S > SP;
- for horseradish root juice, M > MG > SP > S.

#### 3.2.3. Effect of Core-to-Wall Ratio on the Stability of Horseradish-Juice Microcapsules

**Figure 2:**
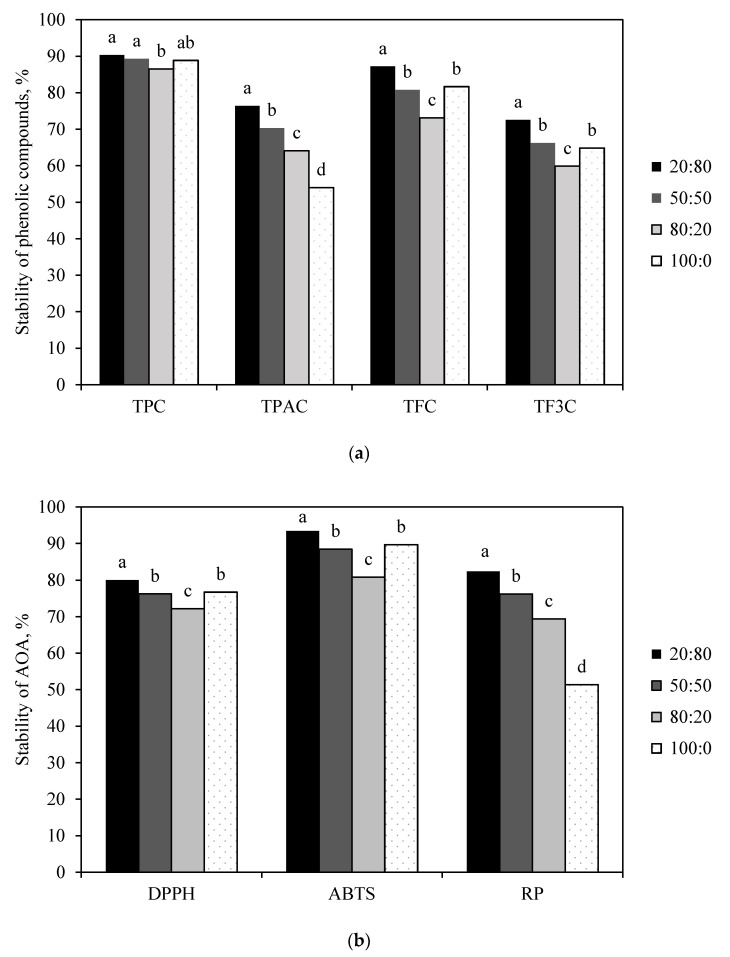
Estimated-marginal-mean plots for the stability of (**a**) phenolic compounds (TPC: total phenolic content; TPAC: total phenolic-acid content; TFC: total flavonoid content; TF3C: total flavan-3-ol content) and (**b**) antioxidant activity (DPPH: 2,2-diphenyl-1-picrylhydrazyl radical activity; ABTS: 2,2′-azino-bis(3-ethylbenzo-thiazoline-6-sulfonic) acid radical activity; RP: reducing power)in the microencapsulated horseradish juice, after four months of storage at room temperature in the dark. The ratio indicates the core-to-wall proportion. ^a–d^ Values with different superscript letters for the same parameter are significantly different (*p* < 0.05) on the basis of Tukey’s multiple-comparison test.

The effect of core-to-wall ratio between plant extract and wall materials in the encapsulation process is a very important factor, significantly affecting the stability of microcapsules during their storage (*p* < 0.05) (Figure 2). A lower core component resulted in higher stability of all tested parameters, which was dependent on the interaction between core-to-wall ratio and wall material. A core-to-wall ratio of 80:20 resulted in lower stability compared to the other tested ratios; for some wall materials and certain tested parameters (TF3C, DPPH^•^, and ABTS^•**+**^), even lower stability than the blank sample was observed. Overall, a core-to-wall ratio of 80:20 was not suitable for the encapsulation of horseradish products. Osamede, Airouyuwa, and Kaewmanee [49] tested two core-to-wall ratios of 1:4 (corresponding to our highest ratio) and 1:9, and they found that storage at 10 °C had no effect on the stability of TPC, but that at 30 °C and 70 °C showed better results for the 1:9 ratio.

The highest stability of TPC, TFC, and TF3C was achieved in the samples encapsulated using MG, reaching an estimated marginal mean for stability of up to 95% for TPC after four months of storage (Table S2, Supplementary Materials). In contrast to TPC, the AOA was lowest in the samples encapsulated with MG. A similar trend in AOA was observed for encapsulated spent coffee, indicating a detrimental effect of both combinations of wall material on AOA; however, in contrast to coffee, the TPC was also lower in the samples with MG [11]. Storage of encapsulated tamarillo powder at 25 °C resulted in reductions in AOA of 11.28% to 49.63%, TPC of 11.32% to 28.16%, and TFC of 14.32% to 35.47% due to the properties of coating material used [8]. The lowest stability (below 60%) was determined for TFC, TPA, and TF3C in individual samples.

The degradation of some biologically active compounds during storage can be attributed to the presence of oxygen in the powders and a higher water activity, leading to a higher rate of decomposition [8].

## 4. Conclusions

The plant part, wall material, and core-to-wall ratio significantly affected the physical parameters of the horseradish-juice microcapsules. Maltodextrin (MD) and maltodextrin/gum Arabic (MG) microcapsules had smaller particles and better solubility but displayed higher hygroscopicity. On the other hand, microcapsules with starch had lower hygroscopicity and solubility but a larger particle size.

The results of this study confirmed the efficiency of microencapsulation for preserving bioactive compounds. However, the plant material, wall material, and core-to-wall ratio significantly affected the retention and stability of the bioactive compounds and the antioxidant activities of microcapsules during their storage. Despite the horseradish-leaf-juice microcapsules containing more bioactive compounds, better retention was seen in the horseradish-root-juice microcapsules. Among the wall materials studied, maltodextrin and maltodextrin/gum Arabic were more effective for the retention of bioactive compounds. In addition, the core-to-wall ratio of 20:80 was more stable during storage. However, more research is required to evaluate the potential application of these microcapsules in food.

## Figures and Tables

**Table 1:** Abbreviations of the tested samples.

Wall Materials	Core to Wall Ratios *					
	Horseradish Leaf Juice			Horseradish Root Juice		
	20:80	50:50	80:20	20:80	50:50	80:20
Maltodextrin (MD)	L20M80	L50M50	L80M20	R20M80	R50M50	R80M20
Maltodextrin/gum Arabic (3:2) (MG)	L20MG80	L50MG50	L80MG20	R20MG80	R50MG50	R80MG20
Soy protein isolate (SPI)	L20P80	L50P50	L80P20	R20P80	R50P50	R80P20
Starch (S)	L20S80	L50S50	L80S20	R20S80	R50S50	R80S20

* Spray-dried horseradish root and leaf juice without wall material was used as a control (blank).

**Table 2:** Technological properties of spray-dried horseradish powders produced with different wall materials.

Materials	Sample Abbreviation	Parameters		
		Particle Size (μm)	Hygroscopicity (g/100 g)	Solubility (%)
Horseradish leaf (L) juice				
Juice (blank)	LB	n.a.	20.99 ± 1.08 ^a^	79.06 ± 0.89 ^g,h^
Maltodextrin (MD)	L20M80	4.24 ± 0.17 ^i^	14.97 ± 0.75 ^d,e,f^	91.02 ± 0.21 ^a^
	L50M50	3.98 ± 0.03 ^j^	16.92 ± 0.82 ^c,d^	88.82 ± 0.47 ^a,b^
	L80M20	3.84 ± 0.07 ^j^	19.25 ± 0.98 ^a,b^	82.53 ± 0.38 ^e,f^
Maltodextrin/gum Arabic (MG)	L20MG80	4.34 ± 0.12 ^i^	12.05 ± 0.60 ^h,i,j^	89.71 ± 1.22 ^a^
	L50MG50	3.77 ± 0.07 ^j,k^	13.40 ± 0.67 ^f,g,h^	84.70 ± 0.63 ^d,e^
	L80MG20	4.20 ± 0.10 ^i^	14.71 ± 0.71 ^e,f,g^	80.74 ± 1.94 ^f,g^
Soy protein isolate (SPI)	L20P80	14.00 ± 1.37 ^b^	8.45 ± 0.39 ^l,m^	59.94 ± 1.23 ^n^
	L50P50	11.50 ± 0.38 ^c^	9.20 ± 0.43 ^k,l,m^	63.44 ± 1.32 ^m^
	L80P20	7.46 ± 0.10 ^f^	10.82 ± 0.57 ^i,j,k^	72.80 ± 0.10 ^i^
Starch (S)	L20S80	9.88 ± 0.84 ^d^	7.46 ± 0.30 ^m^	46.57 ± 0.25 ^r^
	L50S50	14.10 ± 0.50 ^b^	7.93 ± 0.42 ^m^	53.50 ± 0.58 ^p^
	L80S20	9.48 ± 0.25 ^d^	8.29 ± 0.39 ^l,m^	66.60 ± 0.28 ^l^
Horseradish root (R) juice				
Juice (blank)	RB	n.a.	19.73 ± 1.03 ^a,b^	71.87 ± 0.95 ^i,j^
Maltodextrin (MD)	R20M80	3.74 ± 0.02 ^k^	16.01 ± 0.81 ^d,e^	87.05 ± 0.06 ^b,c^
	R50M50	4.01 ± 0.26 ^i,j^	18.89 ± 0.92 ^b,c^	78.52 ± 0.39 ^g,h^
	R80M20	3.89 ± 0.28 ^i,j^	20.89 ± 0.95 ^a,b^	70.30 ± 0.31 ^j,k^
Maltodextrin/gum Arabic (MG)	R20MG80	3.68 ± 0.02 ^k,l^	10.06 ± 0.51 ^j,k,l^	89.56 ± 0.57 ^a^
	R50MG50	3.64 ± 0.12 ^k,l^	12.82 ± 0.64 ^g,h,i^	85.18 ± 0.45 ^c,d^
	R80MG20	3.73 ± 0.08 ^j,k^	14.99 ± 0.60 ^d,e,f^	78.08 ± 0.49 ^h^
Soy protein isolate (SPI)	R20P80	6.57 ± 0.27 ^g,h^	8.19 ± 0.42 ^l,m^	57.03 ± 0.87 ^o^
	R50P50	6.47 ± 0.16 ^h^	9.37 ± 0.47 ^k,l,m^	68.53 ± 0.56 ^k,l^
	R80P20	6.86 ± 0.20 ^g^	10.57 ± 0.53 ^j,k^	67.60 ± 0.55 ^l^
Starch (S)	R20S80	18.40 ± 1.98 ^a^	7.54 ± 0.35 ^m^	28.80 ± 0.56 ^s^
	R50S50	10.30 ± 0.26 ^d^	7.87 ± 0.39 ^m^	46.64 ± 0.04 ^r^
	R80S20	8.84 ± 0.46 ^d,e^	8.04 ± 0.34 ^l,m^	59.80 ± 0.06 ^n^

All data are means ± standard deviation (*n* = 3, dry basis). ^a–n^ Values with different superscript letters in the same column are significantly different (*p* < 0.05) on the basis of Tukey’s multiple-comparison test. n.a.—not analyzed.

**Table 3:** Phenolic composition and antioxidant activity (AOA) of spray-dried horseradish-juice powders with different wall materials.

Materials	Sample Abbreviation	Parameters						
		TPC	TPAC	TFC	TF3C	DPPH^•^	ABTS^•^^+^	RP
Horseradish leaf (L) juice								
Juice (blank)	LB	7232 ± 143 ^a^	3629 ± 92 ^a^	12006 ± 195 ^c^	4550 ± 111 ^i^	148 ± 2 ^a^	1100 ± 20 ^a^	1885 ± 25 ^a^
Maltodextrin (MD)	L20M80	1915 ± 26 ^h^	1664 ± 128 ^g^	2870 ± 186 ^j^	11073 ± 154 ^a^	88 ± 1 ^k^	320 ± 11 ^k,l^	967 ± 2 ^t^
	L50M50	3969 ± 63 ^e^	2357 ± 132 ^d,e^	6686 ± 159 ^g^	9664 ± 177 ^c^	121 ± 2 ^d^	653 ± 20 ^g^	126 ± 1 ^s^
	L80M20	6129 ± 152 ^b^	2925 ± 146 ^b^	11054 ± 192 ^d,e^	4175 ± 191 ^j^	129 ± 2 ^c^	895 ± 14 ^c^	238 ± 5 ^o^
Maltodextrin/gum Arabic (MG)	L20MG80	1867 ± 14 ^i^	2191 ± 127 ^e^	2675 ± 127 ^j^	1823 ± 112 ^r^	98 ± 1 ^h^	305 ± 6 ^l^	291 ± 6 ^k^
	L50MG50	3872 ± 116 ^e^	2960 ± 135 ^b^	6303 ± 254 ^g,h^	2709 ± 28 ^n^	112 ± 2 ^e^	630 ± 8 ^g,h^	459 ± 6 ^f^
	L80MG20	5842 ± 61 ^c^	3602 ± 120 ^a^	10615 ± 153 ^e^	3970 ± 129 ^j^	136 ± 2 ^b^	873 ± 13 ^c,d^	599 ± 6 ^d^
Soy protein isolate (SPI)	L20P80	3103 ± 105 ^f^	2129 ± 151 ^e^	6049 ± 18 ^h^	8334 ± 107 ^e^	107 ± 1 ^f^	544 ± 10 ^i^	70 ± 2 ^w^
	L50P50	4524 ± 75 ^d^	2413 ± 141 ^d^	12851 ± 19 ^b^	9528 ± 124 ^d^	119 ± 1 ^d^	759 ± 14 ^e^	88 ± 3 ^u^
	L80P20	6150 ± 85 ^b^	2927 ± 133 ^b^	18213 ± 19 ^a^	10259 ± 149 ^b^	138 ± 2 ^b^	949 ± 19 ^b^	144 ± 3 ^r^
Starch (S)	L20S80	2282 ± 81 ^g^	2683 ± 125 ^c^	3667 ± 79 ^i^	2374 ± 42 ^o^	109 ± 1 ^f^	377 ± 7 ^j^	324 ± 6 ^j^
	L50S50	4592 ± 47 ^d^	3204 ± 169 ^b^	8079 ± 121^f^	2943 ± 55 ^m^	120 ± 3 ^d^	742 ± 5 ^e,f^	370 ± 7 ^h^
	L80S20	6059 ± 41 ^b^	3729 ± 162 ^a^	10866 ± 265 ^d,e^	3620 ± 135 ^k^	137 ± 2 ^b^	963 ± 19 ^b^	396 ± 8 ^g^
Horseradish root (R) juice								
Juice (blank)	RB	1008 ± 40 ^k,l^	2461 ± 68 ^d^	620 ± 69 ^m^	2173 ± 74 ^p^	96 ± 2 ^h,i^	157 ± 4 ^o^	230 ± 4 ^o,p^
Maltodextrin (MD)	R20M80	321 ± 25 ^p^	1141 ± 107 ^j^	259 ± 10 ^s^	9046 ± 139 ^d^	64 ± 1 ^n^	36 ± 1 ^v^	269 ± 3 ^m^
	R50M50	469 ± 11 ^o^	1257 ± 48 ^h^	307 ± 24 ^r^	6354 ± 75 ^f^	90 ± 1 ^j^	90 ± 2 ^s^	281 ± 5 ^l^
	R80M20	732 ± 18 ^m^	1603 ± 65 ^g^	381 ± 28 ^o,p^	1821 ± 30 ^r^	95 ± 1 ^h,i^	114 ± 1 ^p^	479 ± 6 ^e^
Maltodextrin/gum Arabic (MG)	R20MG80	304 ± 17 ^p^	1542 ± 108 ^g^	208 ± 22 ^t^	1222 ± 50 ^v^	86 ± 2 ^l^	44 ± 1 ^u^	258 ± 3 ^n^
	R50MG50	599 ± 11 ^n^	1930 ± 121 ^f^	420 ± 39 ^o^	1392 ± 62 ^u^	95 ± 2 ^h,i^	90 ± 1 ^s^	603 ± 15 ^d^
	R80MG20	935 ± 75 ^l^	2252 ± 164 ^e^	551 ± 37 ^n^	1714 ± 27 ^s^	102 ± 2 ^g^	108 ± 3 ^r^	838 ± 9 ^b^
Soy protein isolate (SPI)	R20P80	1840 ± 31 ^i^	3127 ± 141 ^b^	568 ± 58 ^m,n^	3194 ± 121 ^l^	95 ± 2 ^h,i^	339 ± 13 ^j^	41 ± 2 ^y^
	R50P50	1407 ± 21 ^j^	2585 ± 138 ^c,d^	834 ± 64 ^l^	4978 ± 73 ^h^	99 ± 1 ^g,h^	223 ± 5 ^m^	55 ± 2 ^x^
	R80P20	1014 ± 32 ^k^	1911 ± 150 ^f^	958 ± 19 ^k^	6050 ± 81 ^g^	101 ± 2 ^g^	195 ± 7 ^n^	75 ± 3 ^v^
Starch (S)	R20S80	325 ± 13 ^p^	1575 ± 115 ^g^	366 ± 34 ^p^	1372 ± 35 ^u^	74 ± 1 ^m^	44 ± 1 ^u^	649 ± 7 ^c^
	R50S50	623 ± 23 ^n^	1619 ± 112 ^g^	490 ± 46 ^n,o^	1514 ± 22 ^t^	94 ± 2 ^i^	73 ± 4 ^t^	352 ± 4 ^i^
	R80S20	852 ± 32 ^l^	2049 ± 116 ^e,f^	521 ± 37 ^n^	1764 ± 19 ^r^	103 ± 2 ^g^	92 ± 4 ^s^	98 ± 2 ^t^

All data are means ± standard deviation (*n* = 3). ^a–v^ Values with different superscript letters in the same column are significantly different (*p* < 0.05) on the basis of Tukey’s multiple-comparison test. TPC: total phenolic content (mg gallic-acid equivalent (GAE)/100 g dry weight (DW)); TPAC: total phenolic-acid content (mg caffeic-acid equivalent (CAE)/100 g DW); TFC: total flavonoid content (mg (+)-catechin equivalent (CE)100 g DW); TF3C: total flavan-3-ol content (mg (+)-catechin equivalent (CE)/100 g DW); DPPH^•^: 2,2-diphenyl-1-picrylhydrazyl radical activity (mmol 6-hydroxy-2,5,7,8-tetramethylchroman-2-carboxylic acid (Trolox) equivalent (TE)/100 g DW); ABTS^•+^: 2,2′-azino-bis(3-ethylbenzo-thiazoline-6-sulfonic) acid radical activity (mmol TE/100 g DW); RP: reducing power (mg ascorbic-acid equivalent (AAE)/100 g DW).

**Table 4:** Average retention (%) of bioactive compounds depending on the applied wall material and the plant part used for juice extraction.

Parameters	Plant Part		Wall Material				
	Roots	Leaves	MD	MG	SPI	S	Blank
TPC	81.58 ^A^	62.77 ^B^	74.93 ^b^	79.94 ^a^	56.40 ^c^	77.43 ^a,b^	75.48 ^b^
TPAC	75.91 ^A^	56.30	72.57 ^a^	74.35 ^a^	47.72 ^c^	69.78 ^b^	67.73 ^b^
TFC	65.99 ^B^	87.38 ^A^	73.71 ^c^	78.84 ^a^	76.23 ^a^	77.97 ^a,b^	75.25 ^b,c^
TF3C	61.08 ^A^	68.90 ^A^	83.53 ^a^	49.22 ^d^	71.27 ^b^	55.93 ^c^	48.62 ^d^
DPPH^•^	78.90 ^A^	49.28 ^B^	69.07 ^a^	67.15 ^b^	59.76 ^c^	60.37 ^c^	61.50 ^c^
ABTS^•^^+^	79.27 ^A^	56.89 ^B^	76.38 ^a^	63.02 ^c^	66.07 ^b^	66.86 ^b^	66.30 ^b^
RP	73.96 ^A^	44.04 ^B^	51.98 ^d^	68.37 ^a^	60.43 ^b^	55.23 ^c^	35.65 ^e^

Data are expressed as the mean of all samples with the same wall material for both horseradish root and horseradish leaf juice (*n* = 18). ^a–^^e^ Values with different superscript letters in the same column are significantly different (*p* < 0.05) on the basis of Tukey’s multiple-comparison test (wall material). ^A–B^ Values with different superscript letters in the same column are significantly different (*p* < 0.05) on the basis of Tukey’s multiple-comparison test (plant part). MD: maltodextrin; SPI: soy protein isolate; MG: maltodextrin/gum Arabic; S: soluble starch; TPC: total phenolic content; TPAC: total phenolic-acid content; TFC: total flavonoid content; TF3C: total flavan-3-ol content; DPPH^•^: 2,2-diphenyl-1-picrylhydrazyl radical activity; ABTS ^•+^: 2,2′-azino-bis(3-ethylbenzo-thiazoline-6-sulfonic) acid radical activity; RP: reducing power.

## Supplementary Materials

The following are available online at https://www.mdpi.com/2304-8158/9/9/1332/s1, Table S1: Retention (%) of bioactives depending on the applied wall material and the plant part used for juice extraction, Table S2: Bioactives and antioxidant activity in the microencapsulated horseradish juice, after four-month storage at room temperature in the dark.
