# Supplementary material for: Microencapsulation of Horseradish (Armoracia rusticana L.) Juice Using Spray-Drying
Source: Foods. 2020 Sep 21;9(9):1332. doi: 10.3390/foods9091332 (PMC7555022; doi:10.3390/foods9091332)
Supplement: Supplementary file 1 [file foods-09-01332-s001.pdf]

**Table S1** Retention (%) of bioactives depending on the applied wall material and the plant part used for juice extraction.

| Materials | Sample abbreviation | Parameters | | | | | | |
|---|---|---|---|---|---|---|---|---|
| | | TPC | TPAC | TFC | TF3C | DPPH$^{\cdot}$ | ABTS$^{\cdot+}$ | RP |
| Horseradish leaf (L) juice | | | | | | | | |
| Maltodextrin (MD) | L20M80 | 69.65 $^{e,f,g}$ | 58.30 $^{g,h}$ | 82.60 $^{a,b,c,d}$ | 98.75 $^{a}$ | 60.45 $^{c}$ | 67.12 $^{e,f,g}$ | 45.01 $^{i,j,k}$ |
| | L50M50 | 66.01 $^{e,f,g,h,i}$ | 51.23 $^{h,i}$ | 88.59 $^{a,b}$ | 89.35 $^{a,b,c}$ | 47.75 $^{d,e}$ | 58.52 $^{g,h,i,j}$ | 31.97 $^{m}$ |
| | L80M20 | 65.49 $^{g,h,i}$ | 47.45 $^{h,i,j}$ | 94.27 $^{a}$ | 61.85 $^{f,g,h}$ | 47.77 $^{d,e}$ | 56.83 $^{g,h,i,j}$ | 34.80 $^{l,m}$ |
| Maltodextrin/gum Arabic (MG) | L20MG80 | 76.63 $^{d,e,f}$ | 90.32 $^{a,b,c}$ | 87.13 $^{a,b,c}$ | 61.98 $^{f,g,h}$ | 57.38 $^{c,d}$ | 60.74 $^{f,g,h,i}$ | 65.89 $^{e,f}$ |
| | L50MG50 | 66.86 $^{f,g,h,i}$ | 74.52 $^{d,e}$ | 86.77 $^{a,b,c}$ | 46.37 $^{j,k,l}$ | 48.19 $^{d,e}$ | 60.14 $^{g,h,i}$ | 56.04 $^{f,g,h}$ |
| | L80MG20 | 62.10 $^{g,h,i,j}$ | 57.73 $^{g,h,i}$ | 90.07 $^{a,b}$ | 58.48 $^{f,g,h,i}$ | 45.24 $^{e}$ | 55.12 $^{h,i,j}$ | 49.95 $^{h,i,j}$ |
| Soy protein isolate (SPI) | L20P80 | 43.62 $^{k}$ | 22.70 $^{l}$ | 69.10 $^{e,f,g,h}$ | 94.94 $^{a}$ | 47.14 $^{d,e}$ | 48.23 $^{j,k}$ | 43.71 $^{j,k,l}$ |
| | L50P50 | 51.53 $^{j,k}$ | 27.74 $^{k,l}$ | 90.25 $^{a,b}$ | 68.12 $^{e,f}$ | 48.93 $^{d,e}$ | 53.96 $^{h,i,j}$ | 32.08 $^{m}$ |
| | L80P20 | 57.77 $^{h,i,j}$ | 38.28 $^{j,k}$ | 94.85 $^{a}$ | 55.09 $^{g,h,i,j}$ | 48.56 $^{d,e}$ | 52.35 $^{i,j}$ | 31.78 $^{m}$ |
| Starch (S) | L20S80 | 65.65 $^{g,h,i}$ | 84.74 $^{c,d}$ | 83.74 $^{a,b,c,d}$ | 80.90 $^{b,c,d}$ | 45.08 $^{e}$ | 55.54 $^{h,i,j}$ | 54.76 $^{g,h,i}$ |
| | L50S50 | 66.14 $^{f,g,h,i}$ | 64.94 $^{e,f,g}$ | 92.78 $^{a,b}$ | 58.16 $^{f,g,h,i}$ | 47.63 $^{d,e}$ | 57.05 $^{g,h,i,j}$ | 42.98 $^{j,k,l}$ |
| | L80S20 | 61.74 $^{g,h,i,j}$ | 57.72 $^{g,h,i}$ | 88.39 $^{a,b}$ | 52.82 $^{h,i,j}$ | 47.20 $^{d,e}$ | 57.13 $^{g,h,i,j}$ | 39.56 $^{k,l,m}$ |
| Horseradish root (R) juice | | | | | | | | |
| Maltodextrin (MD) | R20M80 | 88.60 $^{b,c}$ | 87.56 $^{b,c}$ | 80.94 $^{b,c,d,e}$ | 97.11 $^{a}$ | 77.64 $^{b}$ | 88.64 $^{a,b,c}$ | 39.92 $^{k,l,m}$ |
| | R50M50 | 80.71 $^{c,d}$ | 94.07 $^{a,b,c}$ | 54.01 $^{i,j}$ | 80.08 $^{c,d}$ | 82.91 $^{b}$ | 91.30 $^{a,b}$ | 64.52 $^{e,f,g}$ |
| | R80M20 | 79.12 $^{c,d,e}$ | 96.79 $^{a,b}$ | 41.84 $^{j}$ | 74.05 $^{d,e}$ | 97.92 $^{a}$ | 95.89 $^{a}$ | 95.64 $^{b}$ |
| Maltodextrin/gum Arabic (MG) | R20MG80 | 81.98 $^{b,c,d}$ | 51.44 $^{h,i}$ | 60.59 $^{g,h,j}$ | 51.19 $^{i,j,k}$ | 76.46 $^{b}$ | 38.65 $^{k}$ | 61.18 $^{f,g}$ |
| | R50MG50 | 92.44 $^{a,b}$ | 72.87 $^{f,g}$ | 76.08 $^{c,d,e,f}$ | 39.88 $^{l}$ | 80.48 $^{b}$ | 64.93 $^{f,g,h}$ | 79.73 $^{c,d}$ |
| | R80MG20 | 99.61 $^{a}$ | 99.21 $^{a}$ | 72.40 $^{d,e,f,g}$ | 37.40 $^{l}$ | 95.16 $^{a}$ | 98.55 $^{a}$ | 97.43 $^{a}$ |
| Soy protein | R20P80 | 61.20 $^{g,h,i,j}$ | 54.35 $^{g,h,i}$ | 45.08 $^{j}$ | 91.52 $^{a,b}$ | 53.69 $^{c,d,e}$ | 77.86 $^{c,d,e}$ | 73.89 $^{d,e}$ |

| Materials | Sample abbreviation | Parameters | | | | | | |
|---|---|---|---|---|---|---|---|---|
| | | TPC | TPAC | TFC | TF3C | $DPPH^{\cdot}$ | $ABTS^{\cdot+}$ | RP |
| isolate (SPI) | R50P50 | $56.26^{i,j}$ | $47.17^{i,j}$ | $64.68^{g,h,i}$ | $66.97^{e,f}$ | $61.71^{c}$ | $71.23^{d,e,f}$ | $84.09^{c}$ |
| | R80P20 | $67.99^{f,g,h}$ | $96.11^{a,b}$ | $93.41^{a}$ | $51.01^{i,j,k}$ | $98.54^{a}$ | $92.78^{a}$ | $97.02^{b}$ |
| Starch (S) | R20S80 | $79.33^{c,d,e}$ | $49.66^{h,i}$ | $75.27^{c,d,e,f}$ | $63.60^{e,f,g}$ | $51.95^{c,d,e}$ | $52.70^{i,j}$ | $33.96^{l,m}$ |
| | R50S50 | $91.95^{a,b}$ | $62.56^{f,g}$ | $67.94^{g,h}$ | $41.76^{k,l}$ | $73.42^{b}$ | $80.67^{b,c,d}$ | $62.71^{f,g}$ |
| | R80S20 | $99.78^{a}$ | $99.09^{a}$ | $59.67^{h,i}$ | $38.36^{l}$ | $96.94^{a}$ | $98.07^{a}$ | $97.41^{a}$ |

All data are means ± standard deviation (n = 3). $^{a–m}$ Values with different superscript letters in the same column are significantly different ($p < 0.05$) on the basis of Tukey`s multiple comparison test. TPC: total phenolic content. TPAC: total phenolic acids content. TFC: total flavonoid content. TF3C: total flavan-3-ol content. $DPPH^{\bullet}$: 2,2-diphenyl-1-picrylhydraziyl radical activity. $ABTS^{\bullet+}$: 2,2′-azino-bis(3-ethylbenzo-thiazoline-6-sulfonic) acid radical activity. RP: Reducing power.

**Table S2** Bioactives and antioxidant activity in the microencapsulated horseradish juice, after four-month storage at room temperature in the dark.

| Materials | Sample abbreviation | Parameters | | | | | | |
|---|---|---|---|---|---|---|---|---|
| | | TPC | TPAC | TFC | TF3C | DPPH$^{\cdot}$ | ABTS$^{\cdot+}$ | RP |
| **Horseradish leaf (L) juice** | | | | | | | | |
| Juice (blank) | LB | 6547±207 $^{a}$ | 2511±62 $^{a}$ | 11342±246 $^{a}$ | 3277±81 $^{f}$ | 121±4 $^{a}$ | 1067±37 $^{a}$ | 1097±44 $^{a}$ |
| Maltodextrin (MD) | L20M80 | 1741±52 $^{i}$ | 1484±47 $^{h}$ | 2602±79 $^{h}$ | 6637±191 $^{b}$ | 75±2 $^{f,g,h}$ | 300±8 $^{j}$ | 919±21 $^{b}$ |
| | L50M50 | 3730±145 $^{f}$ | 2198±64 $^{c}$ | 6149±167 $^{f}$ | 5643±167 $^{c}$ | 81±2 $^{f}$ | 596±19 $^{g}$ | 100±3 $^{m}$ |
| | L80M20 | 5374±216 $^{c,d}$ | 2417±100 $^{a,b}$ | 9638±344 $^{c}$ | 3754±119 $^{d,e}$ | 106±3 $^{c,d}$ | 792±22 $^{d}$ | 151±3 $^{l}$ |
| Maltodextrin/gum Arabic (MG) | L20MG80 | 1750±52 $^{i}$ | 1898±47 $^{d,e,f}$ | 2632±79 $^{h}$ | 417±13 $^{l,m,n,o}$ | 64±2 $^{j,k,l,m,n}$ | 261±8 $^{j}$ | 238±4 $^{i}$ |
| | L50MG50 | 3691±94 $^{f}$ | 1927±61 $^{d,e}$ | 6057±244 $^{f}$ | 541±19 $^{l,m,n}$ | 79±2 $^{f,g}$ | 592±19 $^{g}$ | 308±4 $^{h}$ |
| | L80MG20 | 5265±115 $^{d}$ | 2204±47 $^{c}$ | 9021±348 $^{d}$ | 643±13 $^{k,l,m}$ | 101±4 $^{d,e}$ | 858±21 $^{c}$ | 436±11 $^{f}$ |
| Soy protein isolate (SPI) | L20P80 | 2780±88 $^{g}$ | 1735±60 $^{f,g}$ | 2778±96 $^{g,h}$ | 3216±104 $^{f}$ | 81±3 $^{f}$ | 509±13 $^{h}$ | 51±1 $^{n,o}$ |
| | L50P50 | 4115±89 $^{e}$ | 2020±42 $^{d}$ | 6498±167 $^{f}$ | 3583±72 $^{e}$ | 99±3 $^{d,e}$ | 740±23 $^{e}$ | 61±2 $^{m,n,o}$ |
| | L80P20 | 5764±180 $^{a,b}$ | 2396±76 $^{b}$ | 9476±191 $^{c,d}$ | 4022±127 $^{d}$ | 116±4 $^{a,b}$ | 923±24 $^{b}$ | 102±3 $^{m}$ |
| Starch (S) | L20S80 | 2152±68 $^{h}$ | 1790±63 $^{e,f}$ | 3214±96 $^{g}$ | 259±9 $^{o}$ | 74±3 $^{f,g,h,i}$ | 350±14 $^{i}$ | 215±6 $^{i,j,k}$ |
| | L50S50 | 4161±165 $^{e}$ | 1988±59 $^{d}$ | 7321±234 $^{e}$ | 345±12 $^{n,o}$ | 95±2 $^{e}$ | 679±13 $^{f}$ | 304± $^{a}$h9 |
| | L80S20 | 5659±119 $^{b,c}$ | 2395±8b | 10462±420 $^{b}$ | 388±12 $^{m,n,o}$ | 110±6 $^{b,c}$ | 946±38 $^{b}$ | 364±11 $^{g}$ |
| **Horseradish root (R) juice** | | | | | | | | |
| Juice (blank) | RB | 878±31 $^{k,l}$ | 957±24 $^{k,l}$ | 427±19 $^{i}$ | 1252±38 $^{j}$ | 68±2 $^{h,i,j,k,l}$ | 92±2 $^{l,m}$ | 175±5 $^{k,l}$ |
| Maltodextrin (MD) | R20M80 | 255±7 $^{p}$ | 828±22 $^{l,m}$ | 232±5 $^{i}$ | 7109±221 $^{a}$ | 56±2 $^{n}$ | 35±1 $^{n,o}$ | 149±4 $^{l}$ |
| | R50M50 | 377±11 $^{n,o,p}$ | 1197±36 $^{i,j}$ | 243±6 $^{i}$ | 3773±97 $^{d,e}$ | 61±1 $^{l,m,n}$ | 54±1 $^{m,n,o}$ | 219±7 $^{i,j}$ |
| | R80M20 | 646±26 $^{l,m,n}$ | 1556±46 $^{h}$ | 360±10 $^{i}$ | 826±20 $^{k}$ | 76±2 $^{f,g,h}$ | 71±2 $^{m,n}$ | 430±1 $^{a}$f5 |
| Maltodextrin/gum Arabic (MG) | R20MG80 | 294±12 $^{o,p}$ | 769±22 $^{m}$ | 154±5 $^{i}$ | 338±8 $^{n,o}$ | 59±3 $^{m,n}$ | 20±1 $^{o}$ | 182±6 $^{j,k,l}$ |
| | R50MG50 | 581±17 $^{l,m,n,o}$ | 1040±29 $^{j,k}$ | 409±13 $^{i}$ | 446±16 $^{l,m,n,o}$ | 62±2 $^{k,l,m,n}$ | 36±1 $^{n,o}$ | 535±17 $^{d}$ |
| | R80MG20 | 780±23 $^{k,l,m}$ | 1286±32 $^{i}$ | 486±16 $^{i}$ | 687±28 $^{k,l}$ | 70±3 $^{h,I,j,k}$ | 61±2 $^{m,n,o}$ | 699±24 $^{c}$ |

| Materials | Sample abbreviation | Parameters | | | | | | |
|---|---|---|---|---|---|---|---|---|
| | | TPC | TPAC | TFC | TF3C | $DPPH^{\cdot}$ | $ABTS^{\cdot+}$ | RP |
| Soy protein isolate (SPI) | R20P80 | 1603±60 $^{i}$ | 1615±56 $^{g,h}$ | 548±22 $^{i}$ | 1607±58 $^{i}$ | 71±2 $^{g,h,i,j,}$ | 288±9 $^{j}$ | 36±1 $^{o}$ |
| | R50P50 | 1209±40 $^{j}$ | 1451±50 $^{h}$ | 432±14 $^{i}$ | 2204±65 $^{h}$ | 73±2 $^{f,g,h,i}$ | 198±6 $^{k}$ | 47±2 $^{o}$ |
| | R80P20 | 1009±41 $^{j,k}$ | 1034±39 $^{j,k}$ | 489±17 $^{i}$ | 2535±80 $^{g}$ | 80±2 $^{f,g}$ | 125±5 $^{l}$ | 56±2 $^{n,o}$ |
| Starch (S) | R20S80 | 255±7 $^{p}$ | 1096±33 $^{j,k}$ | 160±4 $^{i}$ | 244±9 $^{o}$ | 57±1 $^{n}$ | 30±1 $^{n,o}$ | 488±14 $^{e}$ |
| | R50S50 | 555±17 $^{m,n,o,p}$ | 717±26 $^{m,n}$ | 332±13 $^{i}$ | 264±8 $^{o}$ | 66±2 $^{i,j,k,l,m}$ | 67±2 $^{m,n,o}$ | 248±6 $^{i}$ |
| | R80S20 | 748±26 $^{k,l,m}$ | 583±15 $^{n}$ | 420±12 $^{i}$ | 189±4 $^{o}$ | 69±2 $^{h,i,j,k,l}$ | 89±2 $^{l,m}$ | 90±3 $^{m,n}$ |

All data are means ± standard deviation ($n$ = 3). $^{a–p}$ Values with different superscript letters in the same column are significantly different ($p < 0.05$) on the basis of Tukey's multiple-comparison test. TPC: total phenolic content (mg gallic-acid equivalent (GAE)/100 g dry weight (DW)); TPAC: total phenolic-acid content (mg caffeic-acid equivalent (CAE)/100 g DW); TFC: total flavonoid content (mg (+)-catechin equivalent (CE)100 g DW); TF3C: total flavan-3-ol content (mg (+)-catechin equivalent (CE)/100 g DW); $DPPH^{•}$: 2,2-diphenyl-1-picrylhydrazyl radical activity (mmol 6-hydroxy-2,5,7,8-tetramethylchroman-2-carboxylic acid (Trolox) equivalent (TE)/100 g DW); $ABTS^{•+}$: 2,2′-azino-bis(3-ethylbenzo-thiazoline-6-sulfonic) acid radical activity (mmol TE/100 g DW); RP: reducing power (mg ascorbic-acid equivalent (AAE)/100 g DW).
